# The Causal Association Between Blood Lead and Sleep Disorders: Evidence from National Health and Nutrition Examination Survey and Mendelian Randomization Analysis

**DOI:** 10.1007/s44197-024-00199-4

**Published:** 2024-02-19

**Authors:** Shengnan Chen, Ming Zhang, Weisong Zhang, Xiaolong Shao, Xiaobin Yang, Zhi Yang, Kai Nan

**Affiliations:** 1 Department of Joint Surgery, HongHui Hospital, Xi’an Jiaotong University, Xi’an, 710054 Shaanxi China; 2 Medical Department, Xi’an Jiaotong University, Xi’an, 710048 Shaanxi China; 3 Department of General Practice, Honghui Hospital, Xi’an Jiaotong University, Xi’an, 710054 Shaanxi China; 4 Hongdong County Hospital of Traditional Chinese Medicine, Hongdong, 041600 Shaanxi China

**Keywords:** Sleep disorders, Blood lead, Hypertension, National Health and Nutrition Examination Survey, Cross-sectional study, Mendelian randomization analysis

## Abstract

**Background:**

Poor sleep quality is a global public health concern. This study aimed to identify the risk factors for sleep disorders and clarify their causal effects.

**Methods:**

Data were obtained from the National Health and Nutrition Examination Survey (NHANES) and Mendelian randomization (MR)-Base databases. Baseline characteristics of individuals with and without sleep disorders were compared. A multivariate logistic regression analysis was performed to calculate the effects of each variable on sleep disorders. Causal effects of blood lead levels and hypertension on sleep disorders were assessed using MR analysis.

**Results:**

In total, 3660 individuals were enrolled in the study. The prevalence of self-reported sleep disorders was 26.21%. Serum lead level, serum mercury level, serum retinol level, prevalence of hypertension, and daily vigorous work duration were significantly higher for those in the sleep disorders group than the control group. After adjusting for various covariates, the effects of serum lead and hypertension on sleep disorders were stable from logistic regression models 1–4. MR analysis showed that blood lead levels were causally related to the risk of sleep disorders (odds ratio (OR) = 1.09, 95% confidence interval (CI) 1.01–1.17, *P* = 0.030). There was no causal link between elevated blood pressure and sleep disorders (OR = 0.99, 95% CI 0.94–1.04, *P* = 0.757). Goodness-of-fit tests and sensitivity analyses were used to verify the reliability of the results.

**Conclusions:**

Blood lead is positively and causally associated with an increased risk of sleep disorders. These findings provide a novel perspective regarding sleep protection. Taking effective measures to reduce lead exposure may significantly improve sleep health.

**Supplementary Information:**

The online version contains supplementary material available at 10.1007/s44197-024-00199-4.

## Introduction

Good sleep quality has been widely recognized as an important indicator for maintaining physical health and optimal brain function [1, 2]. The consensus of the American Academy of Sleep Medicine and Sleep Research Society indicates that adults should sleep seven or more hours per night regularly to maintain optimal health [2]. Nonetheless, poor sleep has become a worldwide public health issue in today’s society [3–5]. A meta-analysis conducted on the general population from Netherlands, United Kingdom, and United States revealed that the prevalence of sleep disorders ranges from 9.6 to 19.4% [6]. Therefore, identifying risk factors for sleep disorders may help to alleviate sleep disturbance and improve sleep-related health problems including depression, cognitive dysfunction, Alzheimer’s disease, dry eye disease, obesity, and cardiovascular diseases [7–10].

Sleep health may be influenced by many factors such as sociodemographic status, health status, individual behaviors, psychological conditions, and environmental factors [3–5, 11]. With advancements in industrialization and urbanization, heavy metal pollution has become a global environmental problem. Lead is a well-established environmental neurotoxicant [12]. Previous studies investigating the negative effects of lead have mainly focused on nervous system damage caused by lead exposure. However, the association between blood lead and sleep disorders has not yet received sufficient attention.

A cross-sectional study of 40 female workers exposed to lead showed that most had poor sleep quality [13]. Another cross-sectional study reported a positive association between serum lead levels and sleep duration in premenopausal women, while this association was negative in postmenopausal women [14]. Thus, conflicting findings exist regarding the association between blood lead and sleep disorders in different populations. A cross-sectional study conducted in Mexican children aged 6–8 years showed that increased blood lead levels were associated with decreased sleep duration [15]. Similarly, another study conducted in China showed that elevated blood lead levels among children aged 3–5 years were associated with an increased risk of sleep problems in early adolescence [16]. However, studies in children relied mostly on parental reports, which may lead to inaccurate findings. Meanwhile, owing to the nature of cross-sectional studies, causality cannot be inferred [17]. Therefore, strong evidence supporting the causal link between lead exposure and sleep disorders is still lacking. The same issue also exists in the association between hypertension and sleep disorders. As the pace of life has accelerated and pressure on individuals has increased, researchers have noted a high degree of hypertension and sleep disorders comorbidity [18–20]. However, whether the association is causal remains undetermined. Therefore, to fill these gaps, further research is required to explore causal risk factors for sleep disorders among large-scale populations.

The National Health and Nutrition Examination Survey (NHANES) is a large, nationally representative survey of American civilians that provides comprehensive data on health and nutrition [21]. Data from the NHANES have the potential to facilitate an investigation of the association between risk factors and sleep disorders at the epidemiologic level. Mendelian randomization (MR) is an epidemiological method that uses genetic instrumental variables (IVs) to infer the causal effect of exposure on outcomes [22]. Application of the MR method can help to mitigate the potential challenges of residual confounding and reverse causality. This is because genetic variants are randomly assorted during gamete formation and are not influenced by environmental or lifestyle factors [23, 24]. Therefore, the combined application of NHANES and MR analyses provides reliable and unbiased estimates of the causal effects of risk factors on sleep disorders.

In light of this background, the present study aimed to comprehensively explore risk factors for sleep disorders based on 2017–2018 cross-sectional data of NHANES and clarify the causal effect of risk factors on sleep disorders based on MR analysis. These findings may provide novel insights into the prevention of sleep disorders.

## Methods

### Data Source

The NHANES is a cross-sectional survey that aims to examine the health and nutritional status of the US population [25]. Data for this cross-sectional study were obtained from the 2017–2018 cycles of NHANES. Genome-wide association study (GWAS) datasets for MR analysis were obtained from the National Human Genome Research Institute and European Bioinformatics Institute’s (NHGRI-EBI) GWAS catalog in the MR-Base database [26, 27]. The GWAS IDs for lead levels in the blood, hypertension, and sleep disorders are GCST002831, GCST007707, and finn-b-SLEEP, respectively [28, 29]. Details of the GWAS datasets are given in Supplementary Table 1. No informed consent was required because all data analyzed in this study were publicly available.

### Covariates and Outcome

For the cross-sectional study, covariates including demographic, examination, laboratory, and questionnaire data from the 2017–2018 cycles of the NHANES were defined as covariates. Participants were asked whether they had trouble sleeping via the sleep disorder questionnaire [30], with yes answers indicating a sleep disorders and no answers indicating the absence of a sleep disorders. The answers, “Do not know” and “Refused,” indicated that data regarding the presence or absence of a sleep disorders were missing. For MR analysis, blood lead levels and hypertension were considered exposures, with sleep disorders considered an outcome.

### Statistical Analyses

Baseline characteristics of groups with and without sleep disorders were compared. Shapiro–Wilk test and Levene’s test were used to verify the normality and homogeneity of variance of the baseline data, respectively. The independent-sample *t* test was used to compare the differences in the measurement covariates meeting assumptions of normality and homogeneity of variance. And the data were presented as mean ± standard deviation ($\bar{x }$±s). If normality or homogeneity of variance was not met, differences were assessed using the Wilcoxon rank-sum test, with data presented as the median and interquartile range (IQR). Categorical data were compared using the chi-square test. Multivariate logistic regression was performed to calculate the odds ratio (OR) and 95% confidence interval (CI) for the effects of each variable on sleep disorders. The goodness of fit of the model was evaluated using the Hosmer–Lemeshow test, omnibus test of model, and prediction accuracy.

For MR analysis, single-nucleotide polymorphisms (SNPs) associated with exposures (*P* < 5 × 10^–8^) without linkage disequilibrium were selected as IVs. Inverse variance weighted (IVW), MR-Egger, weighted median, and weighted mode methods were applied to assess the causal effect of blood lead on sleep disorders. MR is based on three conditional assumptions including relevance, independence, and exclusion restriction. Cochran’s Q statistic was used to assess the heterogeneity. The MR-Egger regression intercept was used to examine horizontal pleiotropy. The leave-one-out sensitive analysis was performed to verify the robustness of the results. MR analyses were performed using the TwoSampleMR R package. The present study protocol and details had not been registered.

R studio and SPSS software were used to perform statistical analyses and two-sided *P* < 0.05 was considered as statistically significant.

## Results

### Baseline Data of Included Individuals

A total of 9254 participants were identified from the 2017–2018 cycles of the NHANES. After excluding 2212 participants younger than 18 years old, 87 pregnancies, and 3098 with missing sleep questionnaire data, 3660 individuals were ultimately enrolled in the current study. The overall prevalence of self-reported sleep disorders was 26.21%. Univariate analysis showed that differences in serum lead, hypertension, serum mercury, retinol, and vigorous work between the two groups were statistically significant. The serum lead level, serum mercury level, serum retinol level, prevalence of hypertension, and the proportion of vigorous work in the sleep disorders group were significantly higher than those in the control group. Baseline characteristics of the included participants are presented in Table 1.

**Table 1:** Comparison of baseline characteristics in groups with and without sleep disorders

Variables	Sleep disorders group	No sleep disorders group	*t*/χ^2^/*Z* value	*P* value
Sex (*n*, male/female)	1393/1453	502/509	0.150	0.699
Age (year,$\overline{x }$±s)	50.30 ± 18.73	50.31 ± 18.81	– 0.006	0.995
BMI (kg/m^2^, $\overline{x }$±s)	26.60 ± 8.23	26.35 ± 8.47	0.793	0.428
HR (beats/min, $\overline{x }$±s)	108.27 ± 22.31	108.92 ± 22.92	– 0.333	0.739
SBP (mmHg, $\overline{x }$±s)	122.24 ± 20.94	121.14 ± 20.50	1.275	0.202
DBP (mmHg, $\overline{x }$±s)	67.98 ± 15.99	67.93 ± 15.64	0.086	0.931
Hb (g/L, $\overline{x }$±s)	137.23 ± 15.29	137.32 ± 1.52	– 0.157	0.875
WBC (10^9/L, $\overline{x }$±s)	7.28 ± 2.56	7.27 ± 2.09	0.154	0.878
PLT (10^9/L, $\overline{x }$±s)	259.64 ± 69.41	260.16 ± 72.96	– 0.193	0.847
CRP (mg/L, $\overline{x }$±s)	3.28 ± 0.29	3.27 ± 0.28	– 0324	0.746
HbA1C (%, (M, IQR))	5.50, 1.00	5.50, 1.00	– 1.673	0.094
Ferritin (μg/L, (M, IQR))	80.75, 126.00	74.10, 123.00	– 0.858	0.391
Liver function				
TB (μmol/L, (M, IQR))	6.84, 5.13	7.91, 5.13	– 0.135	0.893
ALT (U/L, (M, IQR))	17.00, 12.00	17.00, 13.00	– 0.835	0.403
AST (U/L, (M, IQR))	19.00, 7.00	19.00, 7.00	– 1.350	0.177
ALB (g/L, (M, IQR))	41.00, 4.00	41.00, 4.00	– 1.127	0.260
Renal function				
SBUN (mmol/L, $\overline{x }$±s)	5.22 ± 2.15	5.22 ± 2.36	0.216	0.829
SCr (μmol/L, $\overline{x }$±s)	78.15 ± 45.40	77.08 ± 42.00	0.610	0.542
SUA (mg/dl, $\overline{x }$±s)	322.74 ± 87.17	318.55 ± 87.61	1.307	0.191
UAlb (mg/L, (M, IQR))	9.10, 13.00	9.80, 15.00	– 1.146	0.252
Serum lipids				
TG (mmol/l, (M, IQR))	0.95, 1	0.94, 1	– 0.277	0.782
TC (mmol/l, $\overline{x }$±s)	4.68 ± 1.04	4.62 ± 1.06	– 0.274	0.784
HDL-C (mmol/l, $\overline{x }$±s)	1.39 ± 0.39	1.34 ± 0.35	0.896	0.371
LDL-C (mmol/l, $\overline{x }$±s)	2.74 ± 0.89	2.82 ± 0.93	– 0.520	0.603
Serum vitamins				
Retinol (µg/dL, $\overline{x }$±s)	1.70 ± 0.52	1.76 ± 0.59	– 2.041	0.041
Vitamin C (umol/L, (M, IQR))	53.25, 38.00	53.10, 35.00	– 1.437	0.151
25-hydroxyvitamin D(nmol/L,$\overline{x }$±s)	67.07 ± 29.35	65.89 ± 26.10	0.377	0.706
Alpha-tocopherol (µg/dL, (M, IQR))	24.38, 10.00	24.85, 11.00	– 0.028	0.978
RBC folate (ng/mL, (M, IQR))	468.00, 213.00	464.00, 220.25	– 0.470	0.639
Serum metals				
Serum calcium (mmol/l, $\overline{x }$±s)	2.33 ± 0.10	2.33 ± 0.10	1.056	0.291
Serum phosphorus (mmol/l, $\overline{x }$±s)	1.18 ± 0.19	1.19 ± 0.20	0.090	0.929
Serum potassium (mmol/l, $\overline{x }$±s)	4.09 ± 0.38	4.08 ± 0.35	1.542	0.123
Serum sodium (mmol/l, $\overline{x }$±s)	140.26 ± 2.70	140.26 ± 2.97	– 1.321	0.187
Serum iron (ug/dL, $\overline{x }$±s)	86.46 ± 36.63	91.04 ± 38.31	– 1.097	0.273
Serum lead (ug/dL, (M, IQR))	0.76, 0.85	0.83, 0.87	– 2.062	0.039
Serum chromium (ug/L, (M, IQR))	0.29, 0.00	0.29, 0.00	– 1.012	0.312
Blood cobalt (ug/L, (M, IQR))	0.16, 0.06	0.16, 0.07	– 0.207	0.836
Serum cadmium (ug/L, (M, IQR))	0.24, 0.00	0.26, 0.00	– 1.575	0.115
Serum mercury (ug/L, (M, IQR))	0.58, 1.00	0.61, 1.00	– 2.606	0.009
Serum selenium (ug/L, $\overline{x }$±s)	189.71 ± 26.59	188.92 ± 26.01	– 0.324	0.746
Serum manganese (ug/L, (M, IQR))	9.55, 4.47	9.82, 4.29	– 0.400	0.689
Disease				
Hypertension (*n*, yes/no)	786/2054	538/470	217.818	< 0.0001
Diabetes (*n*, yes/no)	293/2504	92/899	4.062	0.255
Lifestyle habits				
Alcohol use (*n*, yes/no)	477/1622	179/563	0.604	0.437
Serum cotinine (ng/mL, (M, IQR))	0.03, 1	0.03, 0.00	− 0.934	0.350
Social status				
Vigorous work (*n*, yes/no)	633/2084	260/690	6.667	0.036
Moderate work (*n*, yes/no)	1123/1597	417/541	4.299	0.117
Education level (*n*, Primary school/Middle school/High school/AA degree/College graduate)	234/322/629/662/850	86/113/230/237/289	− 0.308	0.758
Marital status (*n*, never married/ married/widowed or divorced or separated)	318/1337/224	113/459/77	5.817	0.444

*BMI* Body mass index, *HR* Heart rate, *SBP* Systolic blood pressure, *DBP* Diastolic blood pressure, *Hb* Hemoglobin, *WBC* White blood cell, *PLT* Platelet, *CRP* C-reactive protein, *HbA1C* Glycosylated hemoglobin, *TB* Total bilirubin, *ALT* Alanine aminotransferase, *AST* Aspartate aminotransferase, *ALB* Albumin, *BUN* Blood urea nitrogen, *SCr* Serum creatinine, *SUA* Serum uric acid, *UAlb* Urine albumin, *TG* Triglyceride, *TC* Total cholesterol, *HDL*-*C* High-density lipoprotein cholesterol, *LDL-C* Low-density lipoprotein cholesterol, *n* number

### Multivariate Logistic Regression

Variables with statistical significance in the univariate analysis were further included in the multivariate logistic regression (model 1). The crude model was adjusted for demographic factors, social status, and lifestyle habits. Hosmer–Lemeshow goodness-of-fit tests were not significant (*P* > 0.05) indicating a good fit of the multivariate logistic regression models (Supplementary Table 2). All the models were statistically significant (Omnibus *P* < 0.0001). After adjusting for covariates, effects of serum lead level and hypertension on sleep disorders were determined to be stable when applying Models 1–4 (Table 2). Therefore, elevated blood lead and hypertension may be risk factors for sleep disorders.

**Table 2:** Multivariate logistic regression models for sleep disorders

Covariates	Model 1		Model 2		Model 3		Model 4	
	OR (95%CI)	*P*	OR (95%CI)	*P*	OR (95%CI)	*P*	OR (95%CI)	*P*
Serum lead	1.075 (1.006–1.148)	0.032	1.075 (1.006–1.148)	0.032	1.074 (1.005–1.149)	0.036	1.083 (1.003–1.170)	0.042
Hypertension	3.024 (2.534–3.610)	< 0.0001	3.026 (2.535–3.612)	< 0.0001	3.087 (2.574–3.073)	< 0.0001	2.907 (2.340–3.610)	< 0.0001
Serum mercury	1.014 (0.980–1.049)	0.432	1.014 (0.980–1.049)	0.429	1.017 (0.983–1.052)	0.34	1.020 (0.979–1.063)	0.345
Serum retinol	1.177 (1.001–1.384)	0.049	1.176 (1.000–1.384)	0.050	1.156 (0.977–1.366)	0.090	1.113 (0.908–1.363)	0.304
Vigorous work	1.213 (0.991–1.484)	0.061	1.213 (0.991–1.484)	0.061	1.226 (0.997–1.509)	0.053	1.295 (1.012–1.657)	0.040

Model 1 crude model

Model 2 was adjusted for demographic data including age and gender

Model 3 was additionally adjusted for social status including education level and marital status

Model 4: was further adjusted for lifestyle habits including smoking and drinking

### MR Analysis

**Fig. 1:**
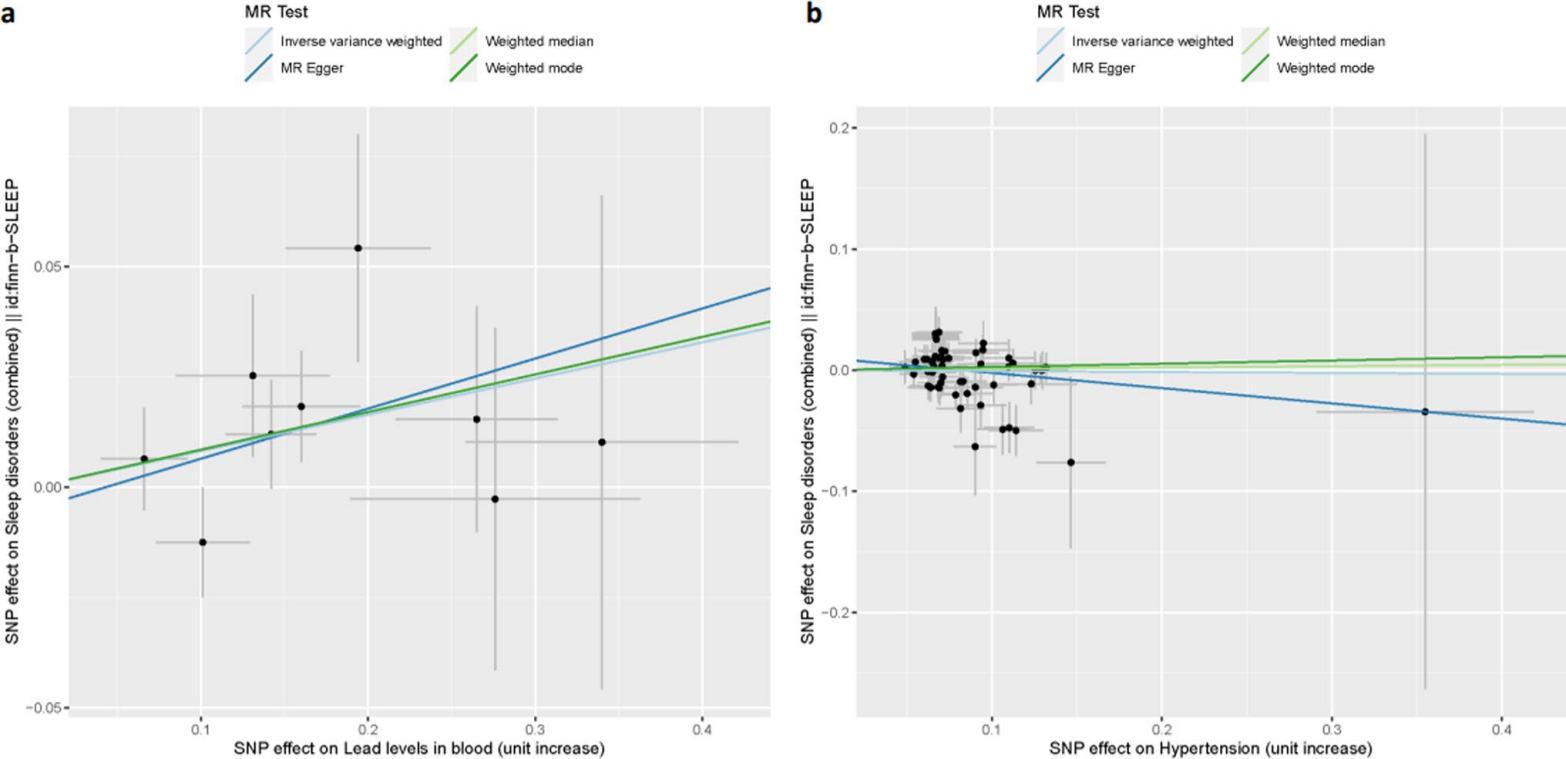
The causal effect of **a** serum lead levels and **b** hypertension on sleep disorders. Colored lines represent the results of MR analysis based on four methods. The slope of the line represents the causal effect of exposure on the risk of sleep disorders

Because a cross-sectional study cannot provide definite information about cause–effect relationships, we attempted to assess the direction of causality based on MR analysis. According to selection criteria, 9 SNPs were selected as IVs to assess the causal relationship between blood lead and sleep disorders. And 56 SNPs were used to assess the causal relationship between hypertension and sleep disorders. The results of the IVW method showed that blood lead levels are causally related to the risk of sleep disorders (OR_IVW_ = 1.09, 95% CI 1.01–1.17, *P* = 0.030). The scatter plot also indicated that the risk of sleep disorders increases with the increase of blood lead (Fig. 1a). No causal link between elevated blood pressure and sleep disorders was identified (OR_IVW_ = 0.99, 95% CI 0.94–1.04, *P* = 0.757) (Fig. 1b).

### Sensitivity Analysis for MR Results

Cochran’s Q and MR-Egger intercept tests confirmed the absence of heterogeneity and horizontal pleiotropy in MR analysis (Supplementary Table 3 and 4). Leave-one-out analysis showed that the results remained stable when SNPs were removed individually, indicating the reliability of MR estimates (Supplementary Figs. 1 and 2).

## Discussion

In the present study, we investigated risk factors for sleep disorders using data of 2017–2018 NHANES participants. Furthermore, we applied the MR approach to assess causal effects of blood lead levels and hypertension on sleep disorders. Results from the observational research showed that serum lead levels were positively related to sleep disorders risk after adjusting for various confounding factors, and the MR analysis further proved that the association was causal. Therefore, high levels of blood lead were directly associated with an increased risk of sleep disorders. Although we found that hypertension was also correlated with sleep disorders, hypertension was not the cause of sleep disorders. To the best of our knowledge, this is the first large-sample study examining the causal association between blood lead and sleep disorders among the real-world population and at the genetic level. Goodness-of-fit tests and sensitivity analyses verified the reliability of our results.

The present study provides direct evidence for the causal effect of blood lead on sleep disorders based on a large-sample cross-sectional study (including adult males and females) and MR analysis. Lead is one of the most toxic metals and potent pollutants [31, 32]. The possible mechanism by which blood lead inducing sleep disorders is that lead may affect the release and reuptake of several neurotransmitters controlled by voltage-gated Ca^2+^ channels to affect the activity of cerebral cortex [33–35]. Fortunately, lead exposure is preventable [36]. Blood levels of lead is a precise estimate of lead exposure [37]. It is generally recognized that environmental pollution is the main cause of human exposure to heavy metals [38]. Pollutants that may expose humans to lead include industrial waste, lead-acid batteries, metal smelting, lead-based paint, leaded gasoline and kerosene, lead-containing water pipes, and contaminated foods [32, 38–42]. Children and pregnant women are more sensitive to lead toxicity than adults [38, 43]. As the subjects in the present study were adults, the impact of lead exposure on sleep disorders may be higher than our estimate. Initiatives aimed at reducing lead exposure, such as strengthening hand hygiene, environmental rehabilitation, and conformity testing of industrial materials and waste, may have significant implications for protecting sleep health. As a result, body energy and immunity can be maintained [44]. We also observed a correlation between hypertension and sleep disorders. Another study that enrolled participants from the 2007–2014 cycles of NHANES also proved that poor sleep patterns were associated with an increased risk of hypertension [20]. However, as is the case with all cross‐sectional studies, associations identified do not necessarily indicate causality [45]. The results of the MR analysis did not support the hypothesis that hypertension causes sleep disorders. This may be because sleep disorders excite the sympathetic nervous system, leading to hypertension [46]. Therefore, further studies are needed to clarify whether sleep disorders are a direct cause of hypertension.

The major strength of this study was the combination of a large-sample cross-sectional investigation and GWAS data. The NHANES is a nationally representative survey and its large sample size is sufficient for providing real-world evidence of the association between blood lead and sleep disorders. MR analysis addresses the inherent effects of residual confounding factors and reverse causality. The combined application of NHANES and MR analyses provides robust and unbiased estimates of the causal effects of blood lead on sleep disorders.

The present study has some noteworthy limitations. First, the diagnosis of sleep disorders was based on self-reported questionnaire. Thus, self-selection, memory, and recall biases may exist. In addition, the populations in the MR analysis were predominantly of European descent; hence, its generalizability to other races may be limited. Another limitation was that we did not explore the specific biological mechanism by which elevated blood lead promote sleep disorders. Further studies are needed to clarify the mechanisms underlying this association.

## Conclusions

Ultimately, we conclude that blood lead is positively associated with an increased risk of sleep disorders, and that this association is causal. These findings provide a novel perspective for the protection of sleep health. Taking effective measures to reduce lead exposure may significantly improve sleep health.

### Supplementary Information

Below is the link to the electronic supplementary material.

Supplementary file1 (DOCX 263 KB)

## Data Availability

The datasets analyzed during the current study are available in the NHANES and MR-Base database.

## Abbreviations

NHANES: National Health and Nutrition Examination Survey
MR: Mendelian randomization
IVs: Instrumental variables
GWAS: Genome-wide association study
OR: Odds ratio
CI: Confidence interval
SNPs: Single-nucleotide polymorphisms
IVW: Inverse variance weighted
BMI: Body mass index
HR: Heart rate
SBP: Systolic blood pressure
DBP: Diastolic blood pressure
Hb: Hemoglobin
WBC: White blood cell
PLT: Platelet
CRP: C-reactive protein
HbA1C: Glycosylated hemoglobin
TB: Total bilirubin
ALT: Alanine aminotransferase
AST: Aspartate aminotransferase
ALB: Albumin
BUN: Blood urea nitrogen
SCr: Serum creatinine
SUA: Serum uric acid
UAlb: Urine albumin
TG: Triglyceride
TC: Total cholesterol
HDL-C: High-density lipoprotein cholesterol
LDL-C: Low-density lipoprotein cholesterol
