# Supplementary material for: The Causal Association Between Blood Lead and Sleep Disorders: Evidence from National Health and Nutrition Examination Survey and Mendelian Randomization Analysis
Source: J Epidemiol Glob Health. 2024 Feb 19;14(2):462–9. doi: 10.1007/s44197-024-00199-4 (PMC11176123; doi:10.1007/s44197-024-00199-4)
Supplement: Supplementary file 1 — Supplementary file1 (DOCX 263 KB) [file 44197_2024_199_MOESM1_ESM.docx]

**Supplementary Table 1. Details of the GWAS datasets**

| GWAS ID | Year | Sample size | Population | Sex |
| --- | --- | --- | --- | --- |
| GCST002831 | 2015 | Australia=2676;  European=2757 | 50.75% European | Males and Females |
| GCST007707 | 2018 | East Asian=22472; European=105253 | 82.40% European | Males and Females |
| finn-b-SLEEP | 2021 | Cases=19155; controls=197545 | European | Males and Females |

**Supplementary Table 2. The goodness-of-fit tests for each model**

|  | Hosmer-Lemeshow test  (*P* value) | Omnibus test of model  (*P* value) | Prediction accuracy (%) |
| --- | --- | --- | --- |
| Model 1 | 0.988 | <0.0001 | 74.6 |
| Model 2 | 0.991 | <0.0001 | 74.5 |
| Model 3 | 0.952 | <0.0001 | 74.6 |
| Model 4 | 0.962 | <0.0001 | 73.8 |

**Supplementary Table 3. Sensitivity analysis for the effect of blood lead on sleep disorders**

| Method | Value | *P* value |
| --- | --- | --- |
| MR-Egger Cochran’s Q test | 3.876 (Q) | 0.275 |
| IVW Cochran’s Q test | 4.638 (Q) | 0.327 |
| MR-Egger regression | -0.0049 (Intercept) | 0.738 |

**Supplementary Table 4. Sensitivity analysis for the effect of hypertension on sleep disorders**

| Method | Value | *P* value |
| --- | --- | --- |
| MR-Egger Cochran’s Q test | 63.57 (Q) | 0.175 |
| IVW Cochran’s Q test | 65.56 (Q) | 0.1558 |
| MR-Egger regression | 0.01 (Intercept) | 0.199 |


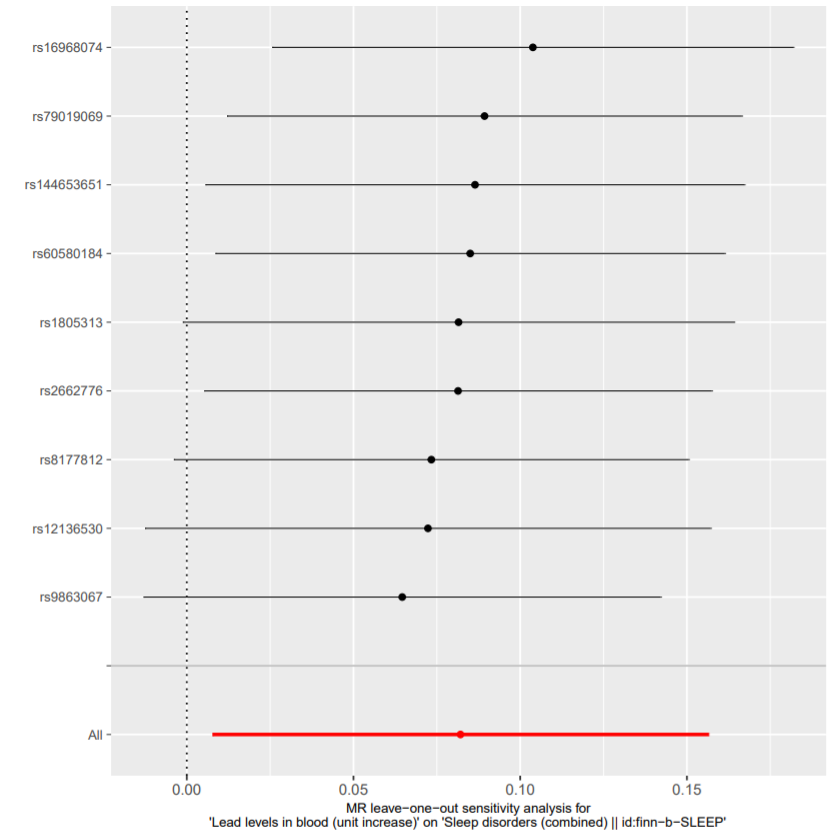


**Supplementary Fig. 1** Leave-one-out analysis for the effect of blood lead on sleep disorders

The black dots are positioned on the right side of the invalid line. This means that removing any of the SNPs will not have a significant impact on the results.


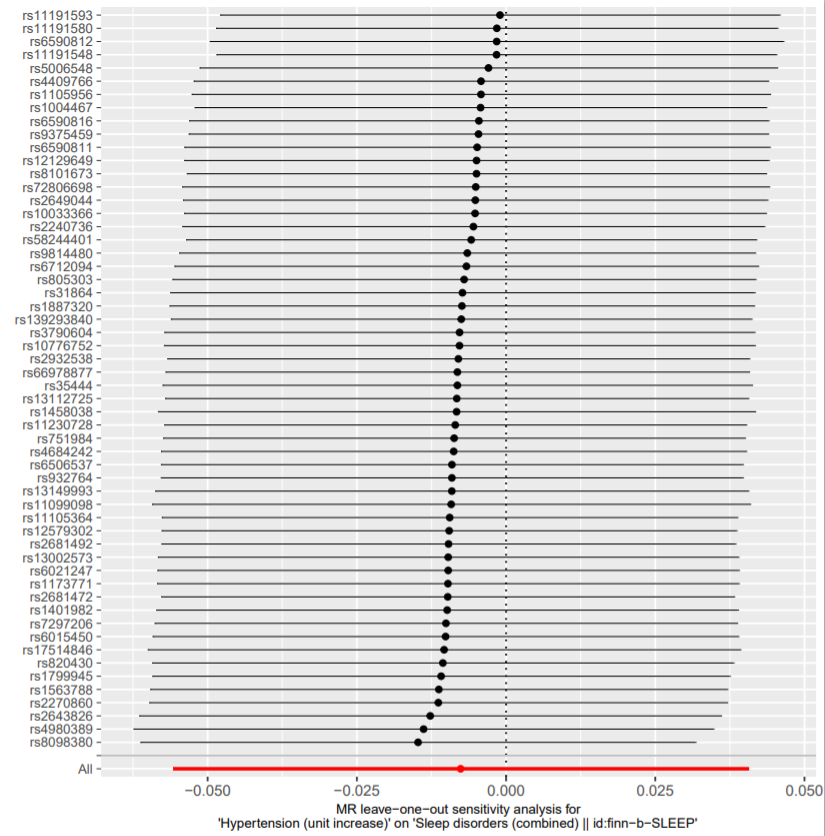


**Supplementary Fig. 2** Leave-one-out analysis for the effect of hypertension on sleep disorders

The black dots are positioned on the left side of the invalid line which indicates that removing any of the SNPs will not have a significant impact on the results.
